# Young Children Display Diurnal Patterns of Salivary IgA and Alpha-Amylase Expression Which Are Independent of Food Intake and Demographic Factors

**DOI:** 10.1155/2019/3687416

**Published:** 2019-01-14

**Authors:** P. W. Lim, S. Nambiar, L. Muhardi, U. H. Abdul Kader, J. Garssen, E. Sandalova

**Affiliations:** ^1^Danone Nutricia Early Life Nutrition, Matrix Building #05-01b, 30 Biopolis Street, Singapore 138667; ^2^Division of Pharmacology, Utrecht Institute for Pharmaceutical Sciences, Faculty of Science, Utrecht University, Utrecht, Netherlands; ^3^Danone Nutricia Early Life Nutrition, Uppsalalaan 12, 3584 CT Utrecht, Netherlands

## Abstract

**Background:**

Salivary alpha-amylase (sAA) and salivary immunoglobulin A (sIgA) have been proposed as biomarkers for research on the mucosal immune system and on stress. Expression of both sAA and sIgA has been described to follow opposing diurnal patterns. This knowledge is crucial for the interpretation of studies using these biomarkers.

**Aim:**

It was hypothesized that sAA and sIgA display diurnal patterns in children and that this is independent of food intake or demographic factors.

**Methods:**

Whole saliva was collected from 78 healthy children (15-39 months old) in the morning and evening for two random nonconsecutive days. The samples have been analysed for sAA and sIgA. The total daily energy, fat, saturated fat, protein, carbohydrate and fibre, mineral, and vitamin consumption were analysed based on the two-day weighed food records collected by the parents.

**Results:**

It was demonstrated that most young children followed the diurnal pattern when sAA increased and sIgA decreased from morning to evening. No correlation was observed between the intake of any of the nutrients and morning or evening values for both salivary proteins. The morning and evening values of sAA and sIgA did not correlate with age, sex, Asian ethnicity, and BMI of the children.

**Conclusion:**

Diurnal patterns of sAA and sIgA exist in healthy young children and are not affected by their nutrient intake, sex, Asian ethnicity, and BMI. Scientists including sIgA and sAA in their research must consider the diurnal pattern that these markers exhibit and design the study accordingly.

## 1. Introduction

More noninvasive approaches for sample collection should be implemented for the treatment and diagnosis of diseases [1–3]. To successfully use noninvasive sampling, a clear understanding of the physiology and biology of chosen biomarkers is necessary [4]. Many of the biomarkers play a role in multiple diseases [5–9]. The differential understating of a marker's biology might rise from its use in different fields.

sIgA is the most abundant immunoglobulin at mucosal surfaces [10]. The main function of sIgA is the opsonisation of invading pathogens [11, 12]. Thus, sIgA has been utilized as a marker for mucosal immunology, as a potential marker for the risk of allergy development [6, 13], and as a marker for research on sports and stress [8, 9, 14–17]. It is clear from the research on mucosal immunology that the microbiota affects the development of sIgA (along with faecal IgA) [18], whereas from stress research it is known that sIgA can display diurnal rhythm [19].

sAA is an enzyme that breaks down carbohydrates and inhibits the growth of pathogens at mucosal surfaces. sAA has been used for stress research and is proposed to be used as a marker for immune status at the oral site [20, 21]. sAA has been described to be lower in the morning and higher in the evening, whereas sIgA is the highest in the morning and lowest in the evening [5].

Most studies have focused on assessing the diurnal pattern of sAA [17, 20, 22–28] and less on the diurnal course of sIgA expression [5, 8, 9, 14, 16, 17, 19]. Interestingly, the time of saliva collection has not been reported in many papers assessing the role of sIgA in allergy and development [6, 7, 13, 29, 30]. Considering reports of a diurnal pattern of sIgA, it is becoming clear that standardizing the time of collection is crucial for the interpretation of the results and should be accounted for in all studies.

IgA's salivary version is produced in salivary glands and actively transported into saliva [31]. The development of IgA's production is influenced by microbial exposure with the gut microbiota playing a key role. In turn, the development and maturation of gut microbiota in young children are affected by the diet [32]. sAA is one the most important salivary enzymes, accounting for 40 to 50% of all the proteins produced in adult saliva. It hydrolyses starch and glycogen into glucose and maltose, initiating the digestive process [20]. A few studies have addressed the expression and diurnal patterns of sAA and the conditions resulting from poor diets, such as obesity or anorexia. The effect of BMI in sAA is not conclusive, as the studies reported either no effect of BMI on sAA secretion and diurnal pattern or a decrease in total or morning sAA with an increase in BMI [22, 33–36].

It is crucial for the correct utilization of these markers to have a common understanding of the factors that influence their expression in relevant body fluid/site, at a relevant age. Understanding when the diurnal patterns are established in childhood and which factors influence them is important for research in children. Multiple factors have been assessed, such as childcare quality, depression, autism spectrum disorder, sleeping patterns, puberty, and mood states [14, 23–25, 36, 37].

However, limited information is available about the effect of the diet and specific nutrients on the levels of sAA and sIgA and on their diurnal patterns, particularly during the developmental stage of childhood [38, 39]. Sevenhuysen et al. (1984) reported that sAA is not affected by starch-containing foods [39]. Filaire and colleagues (2015) observed an overall decrease of sAA secretion in the group of athletes with lower energy, protein, and carbohydrate intakes [38]. No studies have investigated the relationship between sIgA and diet.

In this study, it was hypothesized that sAA and sIgA display diurnal patterns in young children and that this is independent of food intake or demographic factors.

## 2. Materials and Methods

The study included toddlers of 15-39 months of age of Chinese, Malay, or Indian ethnicity in Singapore. The parents of all subjects provided informed and written consent in accordance with the Declaration of Helsinki. The study was approved by the Parkway Independent Ethical Board (PIEC/2015/023). The study has been registered at https://clinicaltrials.gov/show/NCT03138330.

### 2.1. Sample Analysis

The laboratory that performed all the analysis is ISO9001: 2015 certified.

### 2.2. Saliva Collection and Processing

A SalivaBio Children's Saliva (SCS) collection kit (Salimetrics LLC, State College, PA, USA) was given to parents, together with written instructions on how to collect and store the sample. A demonstration was also performed to the parents by the research assistant. The parents were instructed not to perform saliva collection if any symptoms of infection or allergy were observed. The caregiver was requested to provide two clean (that is, before any food or drink was consumed) saliva samples per day over two random nonconsecutive days; the first sample was collected in the morning between 7AM and 9AM and the second sample was collected in the evening between 7PM and 9PM. An SCS swab was inserted in the child's mouth for one minute to absorb sufficient saliva. The saliva swab was then folded into half, inserted in the collection tube, and stored at home in the freezer (approximately -18°C) until it was sent to the laboratory for subsequent saliva processing. Cooler bags were provided for transportation of saliva samples to ensure samples were kept frozen. Upon arrival at the laboratory, samples were thawed and extracted by centrifugation at 3000 rpm for 15 minutes. Subsequently, saliva samples were aliquoted and stored at -20°C until assayed.

### 2.3. Detection of Salivary Total Protein, IgA, and Alpha-Amylase

Protein concentration was obtained following the standard protocol of Pierce™ BCA Protein Assay kit (Thermo Scientific, IL, USA) using bovine serum albumin (BSA) as a standard. The absorbance was detected at 562 nm by a microplate reader (Model: BioTek PowerWave XS2, BioTek Instrument Inc., VT, USA). The intensity of the colour was directly proportional to the protein level in saliva. A four-parameter fitting standard curve was generated using Gen5™ software (BioTek Instrument Inc.) to determine the protein concentration of each saliva sample.

sIgA concentrations were determined by a sandwich ELISA. A 96-well microtiter plate (NUNC-ImmunoTM MaxiSorpTM plates, Nagle Nunc International, CA, USA) was coated at 4°C overnight with mouse anti-human secretory component (sIgA) monoclonal antibody, 5 ug/ml (clone GA-1 Sigma I-6635, Sigma–Aldrich, MO, USA) in 1x Phosphate Buffered Saline (PBS) (1st BASE, Selangor, Malaysia). The following day, the plate was washed three times with PBST (1xPBS, 0.05% Tween-20, Sigma–Aldrich, MO, USA) using an automatic microplate washer model BioTek Elx 405 (BioTek Instrument, VT, USA). Subsequently, the plate was blocked with blocking buffer (1xPBS with 1% BSA, Sigma A-9647) for 1 h at room temperature. A 2-fold serial standard dilution of IgA (purified from human colostrum, I1010, Sigma-Aldrich) ranging from 80 ng/ml to 1.25 ng/ml in blocking buffer and saliva sample were then added to the microplate and incubated for 2 h at room temperature. After two washes, a biotin-conjugated mouse anti-human IgA1/IgA2 monoclonal antibody 0.5 ug/ml (clone G20-359 Pharmingen 35112D) was used for detection of sIgA and incubated for 1h at room temperature. Subsequently, after the third wash, ÉxtrAvidin-HRP conjugate, 1:1000, 2 ug/ml (E2886 Sigma Aldrich) was used to bind the biotin of the anti-secretory component antibody followed by 1h incubation. Following the last wash, 3,3,5,5-tetramethyl benzidine (TMB) substrate (T0440 Sigma-Alrdrich) was added and colouring reaction was stopped using 1N-sulphuric acid after 15 minutes. The absorbance values were measured using the microplate reader (BioTek) at 450nm. A standard curve was constructed with a curve-fitting four-parameter logistic method by using Gen5TM software (BioTek Instrument Inc.). The limit of sIgA detection is 1.25 ng/ml.

sAA was determined using a 96-well alpha amylase kinetic reaction assay kit (Catalog No. 1-1902, Salimetrics LLC, State College, PA, USA). The assay was performed based on the manufacturer's protocol. Briefly, the saliva samples were diluted to 1:200 with the provided diluent. Eight microliters of controls (high and low) and diluted samples was added to the 96-mircrotiter plate followed by adding 320 *μ*L of preheated (37°C) *α*-amylase substrate solution to each well simultaneously. The microplate reader (BioTek Elx 405) was set at 405 nm and 37°C, and absorbance value was read twice, at 1 min and at 3 min. The sAA activity was calculated following the kit instruction: the one-minute readings were subtracted from the three-minute readings and the values were multiplied by the conversion factor. The amount of sAA activity present in the sample is directly proportional to the increase in absorbance detected at 405nm. Results are computed in units per millilitre (U/ml) of alpha-amylase. The limit of sAA detection is 5.1 pg/ml.

The results were rejected if the CV exceeded 20% and the affected sample was re-run. For sIgA the intra-assay CVs ranged from 0.02% to 19% with a mean of final runs 3.3%. For sAA the intra-assay CVs ranged from 0% to 19% with a mean of final runs 3.6%. The mean interassay CV was 12.3% for sIgA and 3.5% for sAA.

### 2.4. Weighed Food Record and Nutrient Analysis

Parents were asked to keep a weighed food record to capture the food and drink intake of their toddler over two nonconsecutive days (one weekday and one weekend day). The full methodology is described in Allan et al [40]. Briefly, participants were given verbal and written instructions on how to complete detailed food records. Templates were provided so they could easily write down the timing of meals, meal type, food preparation details (including brand names of items where applicable), amounts, and the place the item was eaten. Each participant was given digital kitchen scales which could register weights of 1g to 5000g (unnamed; model SF-2012) and they were shown how to tare the weight of plates/cups/bowls before weighing the food and weighing leftovers. Nutrient values were determined with FoodWorks 8 Professional software package (Xyris Software Pty Ltd, Australia). This software linked several national databases available in Australia and allowed new foods to be added (38 generic food items and 19 follow-on and young child formulas were added). For foods specific to Singapore, the Singaporean Health Promotion Board nutrient database [40, 41] was used to create and add new foods to the system (27 items in total). The Composition of Foods Integrated dataset by McCance and Widdowson (revised version) was also consulted [40, 42]. The following macro- and micronutrients were used in the analyses for correlation with sAA and sIgA expression and diurnal patters: total energy (kJ), total protein (g), total fat (g), saturated fat (g), total fibre (g), total sugars (g), and total carbohydrates (g); minerals: calcium (mg), iodine (ug), iron (mg), magnesium (mg), phosphorus (mg), potassium (mg), selenium (ug), sodium (mg), and zinc (mg); and vitamins: beta-carotene (ug), total folate (ug), niacin (mg), retinol (ug), riboflavin (mg), thiamine (mg), total vitamin A (ug), vitamin B12 (ug), vitamin B6 (mg), vitamin C (mg), and vitamin E (mg) [40]. In addition, the participating parents completed a basic demographic questionnaire [40].

### 2.5. Statistical Analyses

Data analysis was performed using GraphPad Prism version 7.05 (GraphPad Software, 2365 Northside Dr Suite 560 San Diego, CA 92108) and IBM SPSS Statistics for Windows, version 23 (IBM Corp., Armonk, N.Y., USA). Online portal http://scistatcalc.blogspot.sg/2013/11/home.html was consulted. Non-normal distribution was assumed based on visual examination of histograms and Shapiro-Wilk test. Therefore, Mann-Whitney tests were used for comparison between groups with usual and unusual diurnal patterns and between weekday and weekend collection. Spearman's rank correlation was used for assessing the relationship between the expression of salivary proteins and nutrient intake. Bonferroni correction for multiple testing was performed for the analysis of day one and day two samples. Box-Cox power transformation was used to transform dependent variables to obtain more normal distributions for regression analysis. For sIgA parameters, the optimal convenient lambda from Box-Cox power transformations is 2; therefore the square transformation was performed. For alpha-amylase parameters, the optimal convenient lambda is 1, so no transformation was performed. Shapiro-Wilk test confirmed that the data were closer to normal distribution after transformation. The multiple regression analysis was performed to assess the relationship between the expression of sAA and sIgA and nutrients as well as for the assessment of the relationship between sAA, sIgA and age, gender, Asian ethnicity, and BMI.

The diurnal pattern was termed usual when the significant difference was observed in the whole population. When a subgroup did not follow the same pattern (increase or decrease throughout the day) the diurnal pattern was termed unusual.

## 3. Results

### 3.1. Study Population

A total of 91 young children aged 15-39 months were recruited in the study for weighed food record analysis; 88 children returned saliva samples. Of these, 78 children (45 boys and 33 girls) provided a full set of required samples. Due to the limited volume of sample for each assay, only 76 paired morning-evening saliva samples for day one and 73 paired samples for day two were analysed for sAA, and 77 and 75 paired samples, for day one and two, respectively, were analysed for sIgA. Seventy-three percent of the participants were of Chinese descent, followed by Malay and Indian with similar sociodemographic characteristics. All participants were generally healthy, without any symptoms at the time of collection. Parents have reported that 17 children had asthma, 16 children had eczema, and 7 had food allergy. Some children were reported to have more than one condition. The total number of children with the parent-reported allergy was 39.

### 3.2. Salivary Alpha-Amylase and Salivary IgA Exhibit Opposing Diurnal Trends

**Figure 1:**
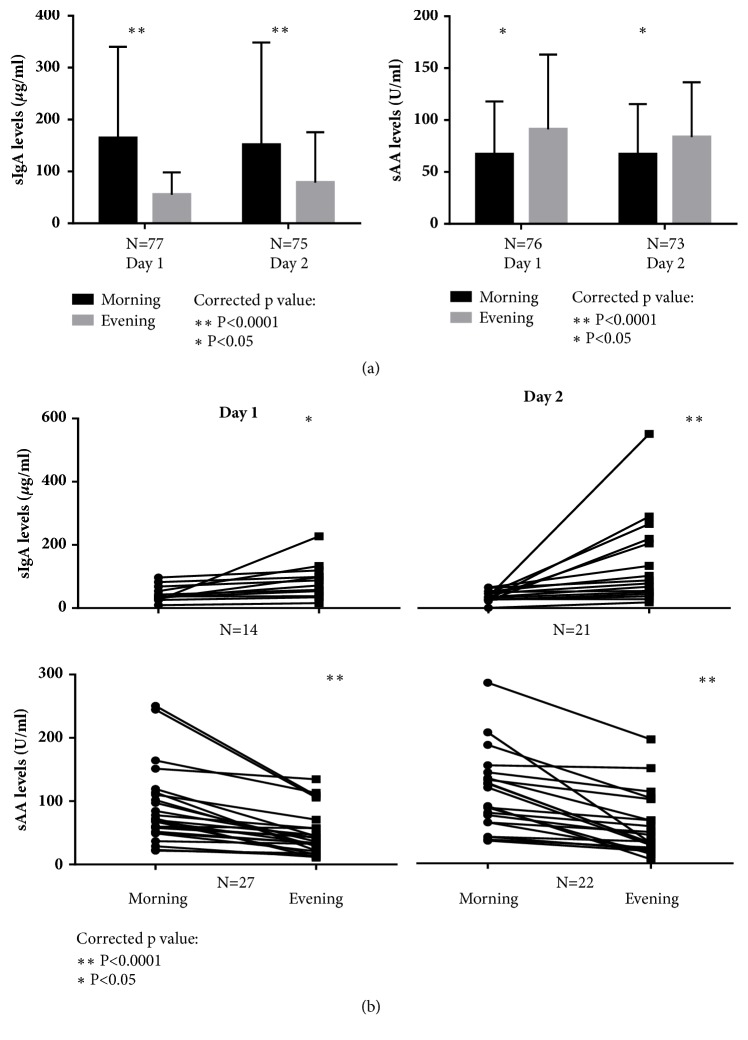
(a)** The diurnal pattern of sAA and sIgA expression in toddlers on day one and day two.** The morning and evening expression levels of sIgA (left) and sAA (right) on day one and day two are shown. The significant difference between morning and evening values is shown with *∗* for <0.05 and *∗∗* for <0.001. The p values were corrected for multiple testing. (b)** Unusual diurnal pattern of sAA and sIgA in toddlers on day one and day two.** The pattern of expression of sIgA (top panel) and sAA (lower panel) was selected and labelled unusual if it differed from general pattern for the whole population shown in (a). A significant difference between morning and evening calculated among the subgroup with unusual diurnal pattern is shown with *∗* for <0.05 and *∗∗* for <0.001. The p values were corrected for multiple testing.

In the current study, the diurnal patterns were demonstrated in young children. sAA level was the lowest in the morning and significantly increased during the day, whereas sIgA level was the highest in the morning and significantly decreased in the evening during both days of saliva collection (Figure 1(a)). This significant difference was observed after correction for multiple testing on two days. It was observed that a proportion of individuals exhibited an unusual diurnal pattern of the sAA or sIgA levels: a few individuals did not show a difference between morning and evening levels, and some showed an opposite pattern with a decreasing trend for sAA and an increasing trend for sIgA within one day (Figure 1(b)). Interestingly, the difference between morning and evening in the subgroup with unusual diurnal patterns was significant in the opposite direction compared to the total group for both sAA and sIgA. The exact time of morning or evening saliva collection and the number of hours between sampling did not affect the diurnal pattern of sAA or sIgA expression (data not shown).

Sex, age, and ethnicity did not explain these unusual patterns (Supplementary Figure 1A). To confirm that there was no effect of sex, the difference between boys and girls in the morning and the evening expression of sAA and sIgA was further examined. However, no significant dissimilarity between boys and girls for both sAA and sIgA expression was observed (Supplementary Figure 2). It was also assessed if a weekend or weekday sampling could affect the sAA or sIgA levels. There were a total of 40 children who collected saliva samples on the weekend on day one and 41 children on day two. The total number of children with an unusual pattern of sAA or sIgA was considered as 100% and the percentage of children with weekend and weekday sampling was calculated. There were no significant differences between weekday and weekend collection for sAA. Interestingly, the sIgA expression on the weekend resulted in less unusual diurnal patterns on day one. Twenty-three percent of children with unusual patterns had weekend samples compared to 77% of children with weekday samples (after Bonferroni correction: p=0.0138). However, there was no significant difference on day two: thirty-one percent of children with unusual patterns in weekend samples and 69% of children in weekday samples (after Bonferroni correction p=0.0688) (Supplementary Figure 1B).

Total protein was measured in all saliva samples. No significant difference was observed between morning and evening samples in the total protein content (Supplementary Figure 3).

Next, we have analysed diurnal patterns in children reported by the parents to have asthma, eczema, and food allergy. Generally, these children displayed the same trend in diurnal patterns as the whole population for both sAA and sIgA. However, the diurnal difference was not always significant, probably due to small sample size for each condition (Supplementary Tables 5-7). The mean values of sAA and sIgA of all the allergic children were compared to the respective mean values of healthy children (Supplementary Table 8). There were no significant differences between allergic and healthy children. Moreover, the differences between morning and evening values for healthy children were significant for both days consistent with the overall population. The diurnal differences for the allergic children were significant for sIgA on both days and for sAA for day two (Supplementary Table 8). There were no differences in the number of children displaying unusual diurnal patterns between allergic and healthy groups (data not shown). Children with atopic conditions did not have any symptoms at the time of saliva collection. Thus, in the current study, the presence of parent-reported allergy did not affect the diurnal patterns of both salivary proteins. However, future studies designed to demonstrate this observation are needed.

### 3.3. sAA and sIgA Diurnal Patterns Are Independent of Nutrient Intake

**Figure 2:**
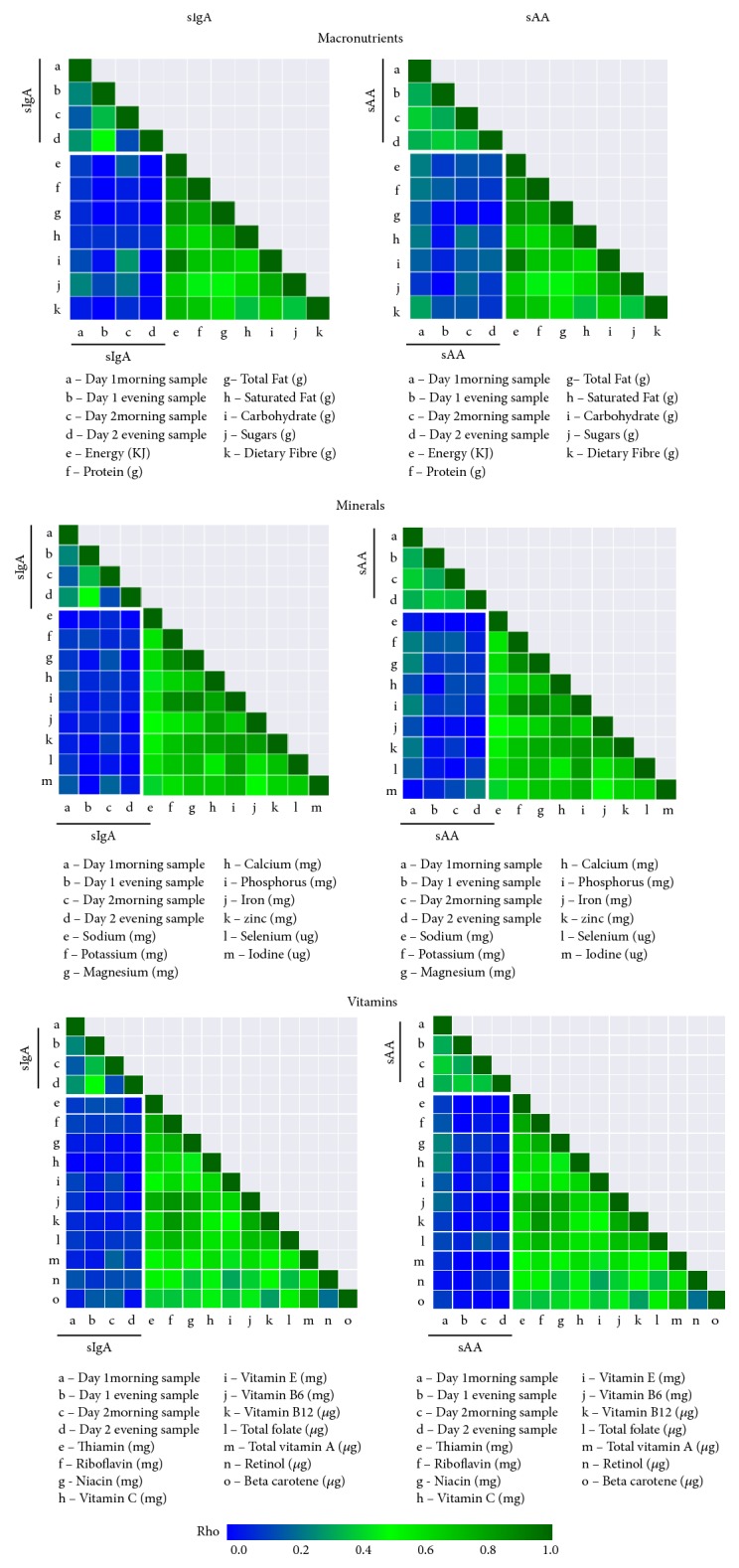
**Correlation of nutrient intake and sAA and sIgA expression in the morning and evening.** Upper panel shows the correlation matrix between sIgA (left), sAA (right), and macronutrients; a to d indicate sIgA/sAA morning and evening on day one and two; e to k indicate the listed macronutrients; middle panel shows the correlation matrix between sIgA (left), sAA (right), and minerals; a to d indicate sIgA/sAA morning and evening on day one and two; e to m indicate the listed minerals; the lower panel shows the correlation matrix between sIgA (left), sAA (right), and vitamins; a to d indicate sIgA/sAA morning and evening on day one and two; e to o indicate the listed vitamins. Spearman's correlation was used and the Rho values were plotted on the graphic scale from 0.0 to 1.0 with the blue colour indicating the lowest and the dark green the highest Rho values.

A two-day weighed food record was used to assess the food intake of the children in the study. The relationships of sAA and sIgA levels and selected macronutrients, minerals, and vitamins were assessed. Correlation matrices were generated for the sAA and sIgA levels in the morning and evening of each day and the nutrients (Figure 2). However, only low nonsignificant correlations were observed between the nutrients and sAA or sIgA.

In addition, multiple regression analysis has been performed for the macronutrients with morning, evening levels of sAA and sIgA as well as with the difference between morning and evening concentrations (day one/day two Δ sAA and Δ sIgA). The adjusted r squared values have ranged from -0.003 to 0.1 for sAA and macronutrients, minerals, and vitamins, and from 0.001 to 0.1 for sIgA and macronutrients, minerals, and vitamins, demonstrating a low level of correlation between the morning, evening values, the morning and evening difference of salivary proteins, and nutrients (Supplementary Tables 1, 2, 3).

Only three parents reported breastfeeding in the weighed food record; however, twenty-five parents reported breastfeeding in the last three months. Thus, it was analysed whether the presence of recent breastfeeding would affect the morning and evening expression of sAA and sIgA. Recent breastfeeding group did not show a significant difference in sAA and sIgA expression with no recent breastfeeding group (data not shown).

### 3.4. Age, Sex, Ethnicity, and Body Mass Index (BMI) Do Not Affect the Morning and Evening Expression of sAA and sIgA

Age, sex, ethnicity, and body mass index (BMI) were assessed for correlation with sAA and/or sIgA expression. The mean age was 22 months. Most of the subjects were Chinese (74%), followed by 18% of Malay and 8% of Indian children. The mean BMI in this study was 17.1 kg/m^2^; the range was 13.0-23.4 kg/m^2^.

BMI did not demonstrate a significant correlation with the levels of sAA. The morning level of sIgA on the second day showed a significant low correlation with BMI (r^2^ 0.2863, p<0.05). However, no significant correlation was observed for the morning sIgA on the first day (r^2^ 0.056). Moreover, there was no significant correlation for the evening values of sIgA on both days (r^2^ =0.005 on day one and r^2^ =0.012 on day two) (Supplementary Figure 4). No significant difference between the levels of expression of both markers by boys and girls was observed (Supplementary Figure 2). Multiple regression was performed for morning and evening levels of sAA and sIgA on days one and two, as well as for the difference between the morning and the evening values of sAA and sIgA (day one/day two Δ sAA and Δ sIgA). The morning and evening levels of sAA did not show significant correlation with these factors on both days; the adjusted r squared ranged from 0.006 to 0.07 (Supplementary Table 4). The morning and evening levels of sIgA also showed a low nonsignificant level of correlation with age, sex, ethnicity, and BMI. The adjusted r squared ranged from 0.003 to 0.09 (Supplementary Table 4).

### 3.5. sAA Expression Does Not Correlate with sIgA Expression

**Figure 3:**
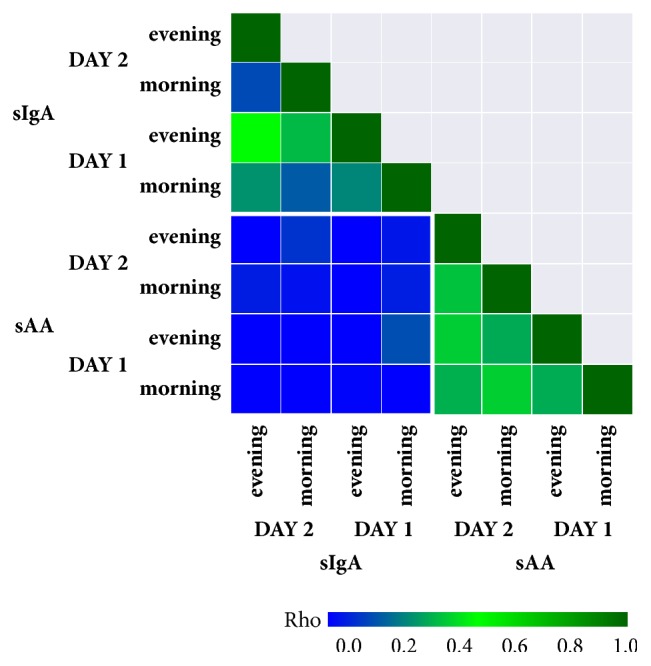
**Correlation matrix of morning and evening expression of sAA and sIgA. **Spearman's correlation was used and the Rho values were plotted on the graphic scale from 0.0 to 1.0 with the blue colour indicating the lowest and the green the highest Rho values.

A correlation matrix on Figure 3 shows that there was only a very low correlation between sAA and sIgA levels either in the morning (day one: rho = -0.029; day two: rho = -0.043) or in the evening (day one: rho = -0.096; day two: rho =-0.006). No significant correlation between the morning and evening differences in sAA and sIgA levels was observed on both days (day one: rho = -0.205; day two: rho = -0.164).

## 4. Discussion

A better understanding of biology is necessary for successful implementation of noninvasive biomarkers. Salivary biomarkers are a good example of a noninvasive approach.

This study has shown that salivary sAA and sIgA follow opposing diurnal patterns in healthy toddlers. sAA is low in the morning and increases during the day, whereas sIgA is high in the morning and decreases in the evening. There are important implications for this phenomenon. When using these salivary biomarkers in research, time of collection needs to be standardized. Even though it has been shown that sIgA displays diurnal variation [8, 16, 17, 19, 43], it is not always taken into account in other studies [6, 7, 30, 44]. Thus, the diurnal pattern of sIgA is not yet widely accepted and accounted for in all research areas. Therefore, it is crucial to demonstrate diurnal patterns of sIgA expression in children to facilitate the appropriate interpretation of the results for future studies. sAA was also included in our study as a comparison to sIgA. For sAA, more studies have demonstrated the diurnal pattern [17, 22–24, 28, 34, 45]. Yet the age when these patterns are established is not evident. It has been shown that the level of sAA in the newborn is very low and demonstrates a steady increase during the first six months of life [39]. One study stated that the time of sample collection in 24-month-old children did not affect the expression of sAA [46]. Several studies have shown the diurnal pattern of sAA in children older than 4 years of age [34, 47–49].

Interestingly, in this study, a proportion of children did not show the same patterns consistently. The samples were collected on two random nonconsecutive days. Young children exhibiting an unusual diurnal pattern for sIgA and sAA expression largely did not overlap and differed from one day to another. It has been previously demonstrated that some individuals display no circadian difference and even reverse trends [24]. In addition, it has been reported that the interindividual, as well as intraindividual differences in sAA expression, could be largely accounted for by genetic and environmental factors. Moreover, how each subject responds to environmental triggers could be also genetically determined [45, 50].

Children's diet, a variable environmental factor, could also contribute to inter- and intraindividual variations of the levels of sAA and sIgA. In this study, information on diet and saliva samples was collected during the same time frame (within one week). However, macro- and micronutrient intake did not influence sAA and sIgA expression in young children. This could be explained by the fact that no extreme dietary habits were observed. The very low adjusted r squared values were observed, indicating that there is a limited possibility that interaction between the nutrient intakes and the expression of sAA or sIgA exists. However, it is possible that when the children's diet changes as they grow older, and the variety increases, then the effect of nutrient intake on sAA and sIgA expression could be observed.

The BMI of the children were within normal ranges, and the effect of BMI of diurnal patterns of sAA and sIgA was not observed (either as a standalone factor or in multiple regression analysis including other demographic factors). Interestingly, sIgA showed a weak significant correlation with BMI on the second day (r^2^=0.286); however, this association disappeared when age, sex, and ethnicity were applied in multiple regression analysis. It is also possible that if the population had a greater range of BMI, then a stronger interaction with diurnal patterns of sIgA may have been detected. It has been previously reported for sAA that its diurnal pattern is linked to the adiposity of preschool-aged children from low-income families [34]. This study included overweight and obese children and therefore could demonstrate the difference between the groups. Moreover, the children were exposed to chronic life stressors such as poverty, which also affected the sAA expression [34].

In this study, child's sex, age, and ethnicity did not show an effect on the diurnal pattern of both sAA and sIgA and could not explain the unusual patterns observed. Sex differences for a diurnal pattern of sAA have been observed in some studies [22, 28]; however, these differences were reported only in adults, where men showed a lower increase in sAA compared to women.

To our knowledge, no study has examined the effect of ethnicity on the diurnal patterns of sAA and sIgA expression. All subjects in our study were of Chinese, Malay, or Indian ethnicity. Indian and Malay children were too few to be analysed separately. Thus, only Chinese and non-Chinese ethnic groups were analysed. This study has confirmed the presence of diurnal patterns of sAA and sIgA expressions in Southeast Asians and did not demonstrate any influence of Chinese ethnicity.

It was possible that the sleep patterns of individual children could affect the diurnal patterns of sAA and sIgA. Young children of this age could be taking one or multiple naps during the day [51, 52], which could alter the expression of these salivary markers. This study did not aim to demonstrate the dependence of sAA and sIgA on sleeping patterns, but only to show dependence on the sample collection time. No sleeping diaries were collected and thus it cannot be excluded that the observed unusual diurnal patterns were due to daily naps of the children.

A study showed a different sIgA pattern during weekday and weekend in young pre-schoolers [36] where a declining daily pattern of sIgA was only evident on weekend day. In this study, it was suspected that the weekend versus weekday sampling could affect the diurnal pattern of sAA and sIgA. sAA expression did not substantially vary according to the weekend or weekday sampling in the children who exhibited an unusual diurnal pattern. For sIgA, day one being the weekend day seemed to result in a more usual diurnal pattern, which was in line with Watamura's study [36]. On day two, the difference between weekend and weekday sampling for sIgA was also significant but did not survive the correction for multiple testing. A future study designed to assess this observation would be needed to confirm these findings.

Saliva collection from young children can be unexpectedly challenging and time-consuming [4]. One study among young children which collected blood, urine, and saliva samples showed that the completion of blood collection was the highest, followed by urine and saliva as the least [53]. Bottcher and colleagues have also noted that in their study the saliva samples were collected relatively easy for 3, 6, and 12 months; however, many children heavily protested during the 24-month collection time point [6]. In the current study, there were also difficulties in collecting saliva samples. Children refused to open their mouths and pushed the swabs out. The volume of sample was limited, indicative of the struggle that the parents faced when performing sample collection. The struggle of the parent to collect samples could have induced stress to the child which also could have affected the sAA and sIgA diurnal patterns. Altogether, this would be important to consider when designing future studies that aim at collecting saliva from young children.

The diurnal pattern of sIgA is known to show the same trend as cortisol but an opposing trend with sAA [5, 8, 19]. In this study, only low nonsignificant correlations between morning and evening levels of sAA and sIgA were observed. Moreover, the difference between the morning and evening levels (delta sAA and delta sIgA) only demonstrated low, nonsignificant correlation. This is not surprising as it is known that sAA reflects the diurnal changes in the autonomous nervous system, whereas the transport of sIgA into saliva may reflect the HPA axis activation [8, 20, 54].

## 5. Conclusion

The diurnal patterns of sAA and sIgA were demonstrated in Asian young children and were independent of nutrient intake, sex, age, or Chinese ethnicity. sAA was low in the morning and increased in the afternoon and sIgA was high in the morning and decreased during the day. A proportion of children did not show the same diurnal patterns as an overall population. Future clinical studies which assess sAA and sIgA expression must take diurnal patterns into account.

## Data Availability

All the data used to support the findings of this study are available from the corresponding author upon request.

## Supplementary Materials

Supplementary Materials Supplementary material includes 4 figures and 8 tables.** Supplementary Figure ****1:** effect of sex, age, and ethnicity (A) and weekend versus weekday (B) sampling on sAA and sIgA unusual diurnal pattern in toddlers.** Supplementary Figure ****2:** expression of sAA and sIgA in boys and girls in the morning and evening for two days.** Supplementary Figure ****3:** total protein expression in the saliva samples of morning and evening for day one and day two.** Supplementary Figure ****4:** correlation of BMI of the children with their morning and evening expression of sIgA and sAA.** Supplementary Table ****1**: adjusted R-square values for multiple regression model with predictors: Energy, Total Fat, Saturated Fat, Sugar, Carbohydrate, Protein, and Dietary Fiber. Δ sAA/Δ sIgA was derived by subtracting sAA am/ sIgA am from sAA pm/ sIgA pm values.** Supplementary Table ****2:** adjusted R-square values for multiple regression model with predictors: Beta-carotene, Total Folate, Niacin, Retinol, Riboflavin, Thiamine, Vitamin A, Vitamin B12, Vitamin B6, Vitamin C, and Vitamin E. Δ sAA/Δ sIgA was derived by subtracting sAA am/ sIgA am from sAA pm/ sIgA pm values.** Supplementary Table ****3**: adjusted R-square values for multiple regression model with predictors: Calcium, Iodine, Iron, Magnesium, Phosphorus, Potassium, Selenium, Sodium, and Zinc. Δ sAA/Δ sIgA was derived by subtracting sAA am/ sIgA am from sAA pm/ sIgA pm values.** Supplementary Table ****4**: adjusted R-square values for multiple regression model with predictors: BMI, Ethnicity, Age, and Gender. Δ sAA/Δ sIgA was derived by subtracting sAA am/ sIgA am from sAA pm/ sIgA pm values.** Supplementary Table ****5**: sIgA and sAA expression (in pg/ml) in morning and evening on two days in children who were reported by the parents to have eczema (N=16).** Supplementary Table ****6**: sIgA and sAA expression (in pg/ml) in morning and evening on two days in children who were reported by the parents to have asthma (N=17).** Supplementary Table ****7**: sIgA and sAA expression (in pg/ml) in morning and evening on two days in children who were reported by the parents to have food allergy (N=7).** Supplementary Table ****8:** mean morning and evening values of sIgA and sAA expression for healthy children and children with parent-reported allergy.
