# Supplementary material for: Young Children Display Diurnal Patterns of Salivary IgA and Alpha-Amylase Expression Which Are Independent of Food Intake and Demographic Factors
Source: Biomed Res Int. 2019 Jan 14;2019:3687416. doi: 10.1155/2019/3687416 (PMC6348797; doi:10.1155/2019/3687416)
Supplement: Supplementary Materials — Supplementary material includes 4 figures and 8 tables. Supplementary Figure 1: effect of sex, age, and ethnicity (A) and weekend versus weekday (B) sampling on sAA and sIgA unusual diurnal pattern in toddlers. Supplementary Figure 2: expression of sAA and sIgA in boys and girls in the morning and evening for two days. Supplementary Figure 3: total protein expression in the saliva samples of morning and evening for day one and day two. Supplementary Figure 4: correlation of BMI of the children with their morning and evening expression of sIgA and sAA. Supplementary Table 1: adjusted R-square values for multiple regression model with predictors: Energy, Total Fat, Saturated Fat, Sugar, Carbohydrate, Protein, and Dietary Fiber. Δ sAA/Δ sIgA was derived by subtracting sAA am/ sIgA am from sAA pm/ sIgA pm values. Supplementary Table 2: adjusted R-square values for multiple regression model with predictors: Beta-carotene, Total Folate, Niacin, Retinol, Riboflavin, Thiamine, Vitamin A, Vitamin B12, Vitamin B6, Vitamin C, and Vitamin E. Δ sAA/Δ sIgA was derived by subtracting sAA am/ sIgA am from sAA pm/ sIgA pm values. Supplementary Table 3: adjusted R-square values for multiple regression model with predictors: Calcium, Iodine, Iron, Magnesium, Phosphorus, Potassium, Selenium, Sodium, and Zinc. Δ sAA/Δ sIgA was derived by subtracting sAA am/ sIgA am from sAA pm/ sIgA pm values. Supplementary Table 4: adjusted R-square values for multiple regression model with predictors: BMI, Ethnicity, Age, and Gender. Δ sAA/Δ sIgA was derived by subtracting sAA am/ sIgA am from sAA pm/ sIgA pm values. Supplementary Table 5: sIgA and sAA expression (in pg/ml) in morning and evening on two days in children who were reported by the parents to have eczema (N=16). Supplementary Table 6: sIgA and sAA expression (in pg/ml) in morning and evening on two days in children who were reported by the parents to have asthma (N=17). Supplementary Table 7: sIgA and sAA expression (in pg/ml) in morning [file 3687416.f1.pdf]

Young children display diurnal patterns of salivary IgA and alpha-amylase expression which are independent of food intake and demographic factors

Lim P.W.1, Nambiar S.1, Muhardi L.1, Abdulkader U.1, Garssen J. 2,3, Sandalova E.1,2 *

Supplementary materials

Includes 4 figures and 8 tables.

Supplementary Figure 1
Effect of sex, age and ethnicity (A) and weekend versus weekday (B) sampling on sAA and sIgA unusual diurnal pattern in toddlers.
Supplementary Figure 2
Expression of sAA and sIgA in boys and girls in the morning and evening for two days.
Supplementary Figure 3
Total protein expression in the saliva samples of morning and evening for day one and day two.
Supplementary Figure 4
Body mass index of the children and sIgA (left) and sAA (right) expression in the morning and evening for both days.

Supplementary Figure 1

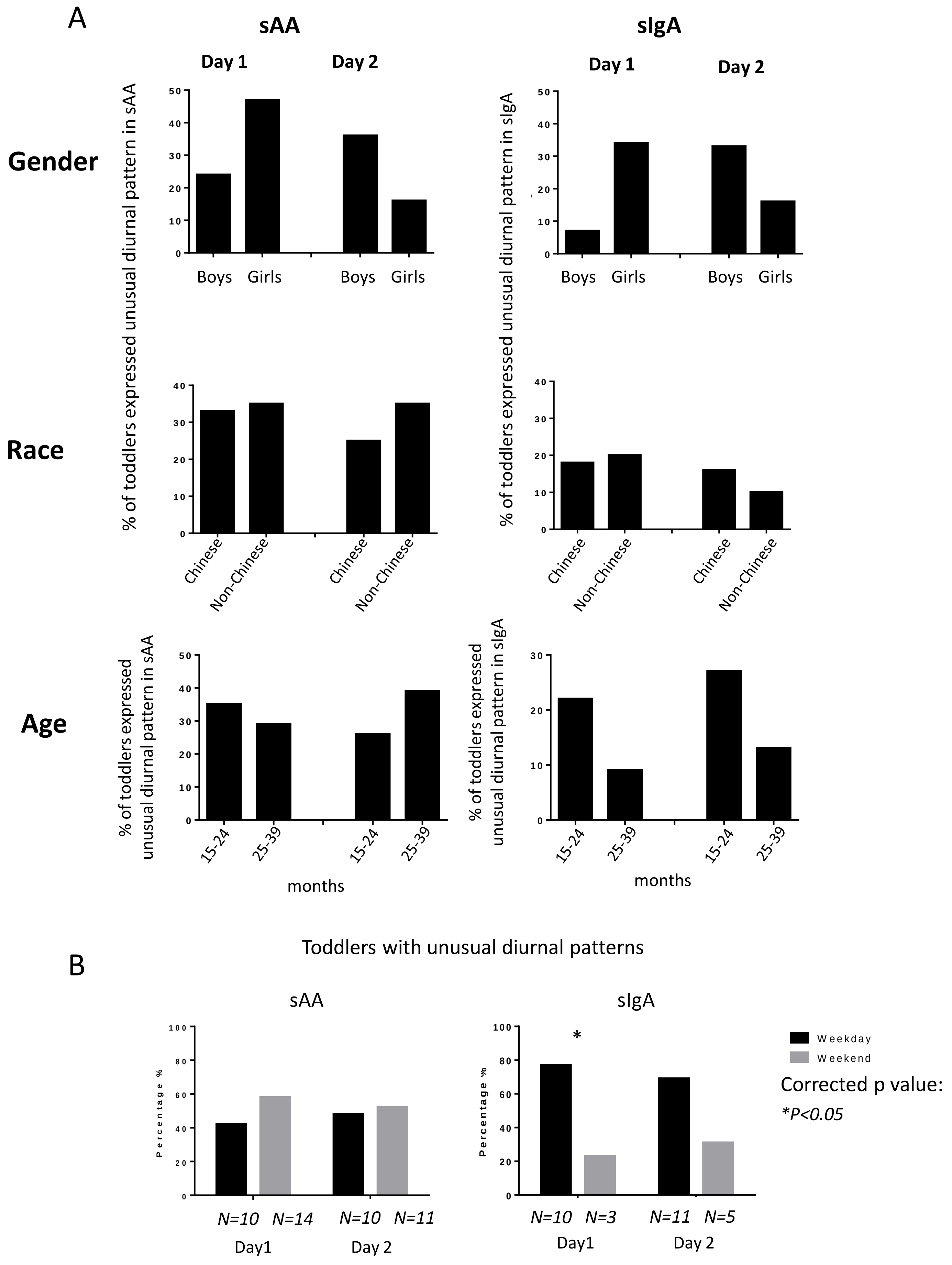

Supplementary figure 2

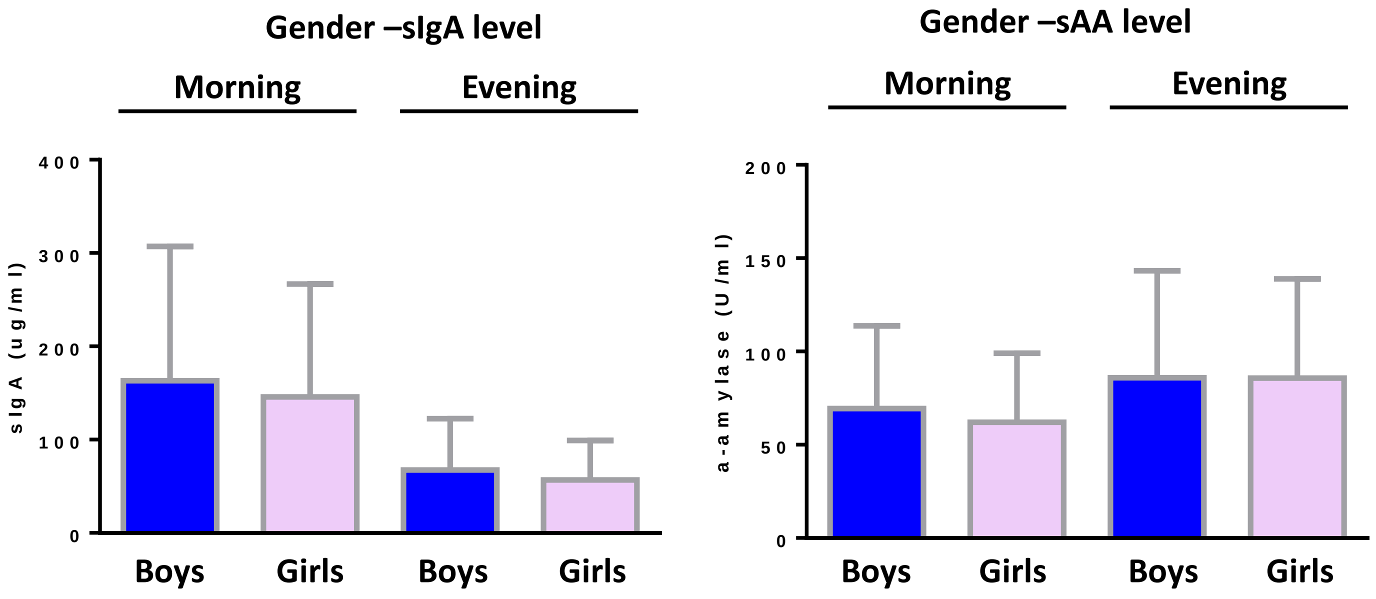

Supplementary Figure 3

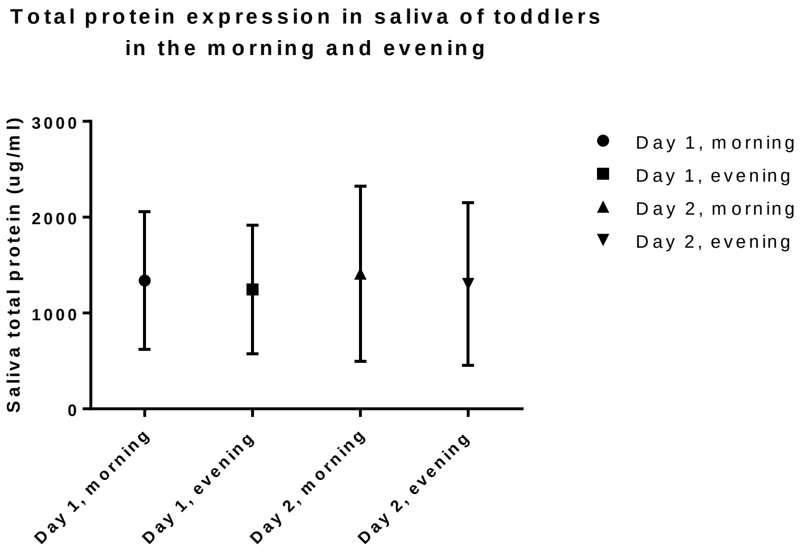

Supplementary Figure 4

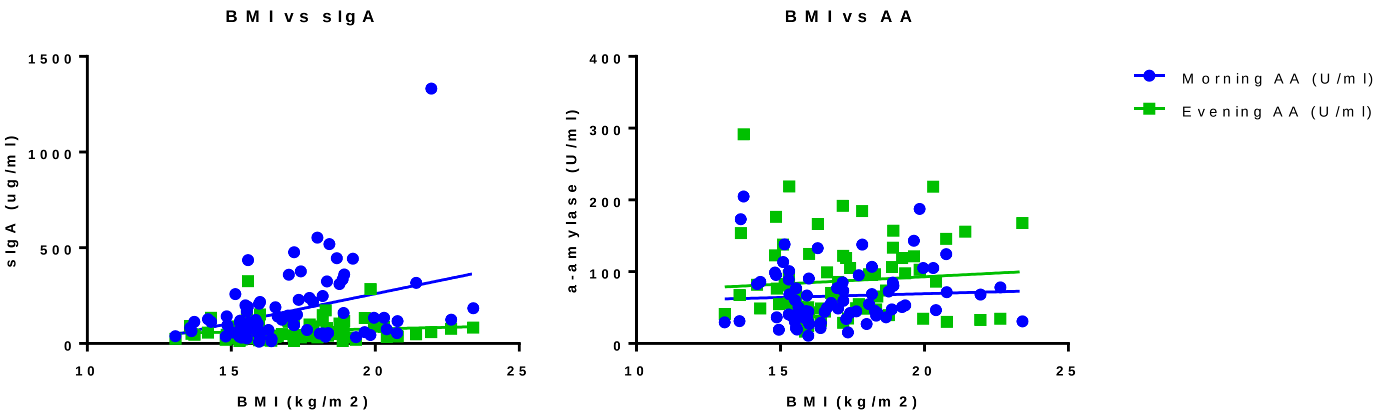

Supplementary Table 1. Adjusted R-square values for multiple regression model with predictors: Energy, Total Fat, Saturated Fat, Sugar, Carbohydrate, Protein, Dietary Fiber. Δ sAA / Δ sIgA was derived by subtracting sAA am/ sIgA am from sAA pm/ sIgA pm values.

Predictors	Dependent Variables	adjusted R square
7 Macronutrients	Day1 sAA am	0.053
	Day1 sAA pm	-0.003
	Day2 sAA am	0.060
	Day2 sAA pm	-0.031
	Day1 sIgA am	0.068
	Day1 sIgA pm	-0.045
	Day2 sIgA am	0.020
	Day2 sIgA pm	-0.057
	Day1 Δ sAA	-0.037
	Day2 Δ sAA	-0.055
	Day1 Δ sIgA	0.010
	Day2 Δ sIgA	-0.004

Supplementary Table 2. Adjusted R-square values for multiple regression model with predictors: Beta-carotene, Total Folate, Niacin, Retinol, Riboflavin, Thiamine, Vitamin A, Vitamin B12, Vitamin B6, Vitamin C, and Vitamin E. Δ sAA / Δ sIgA was derived by subtracting sAA am/ sIgA am from sAA pm/ sIgA pm values.

Predictors	Dependent Variables	Adjusted R square
11 Micronutrients	Day1 sAA am	-0.011
	Day1 sAA pm	-0.005
	Day2 sAA am	0.100
	Day2 sAA pm	0.051
	Day1 sIgA am	-0.007
	Day1 sIgA pm	-0.062
	Day2 sIgA am	-0.104
	Day2 sIgA pm	0.058
	Day1 Δ sAA	-0.044
	Day2 Δ sAA	-0.050
	Day1 Δ sIgA	0.000
	Day2 Δ sIgA	-0.081

Supplementary Table 3. Adjusted R-square values for multiple regression model with predictors: Calcium, Iodine, Iron, Magnesium, Phosphorus, Potassium, Selenium, Sodium, and Zinc. Δ sAA / Δ sIgA was derived by subtracting sAA am/ sIgA am from sAA pm/ sIgA pm values.

Predictors	Dependent Variables	Adjusted R square
9 Minerals	Day1 sAA am	0.02
	Day1 sAA pm	-0.062
	Day2 sAA am	0.028
	Day2 sAA pm	0.043
	Day1 sIgA am	-0.068
	Day1 sIgA pm	0.068
	Day2 sIgA am	-0.023
	Day2 sIgA pm	-0.011
	Day1 Δ sAA	-0.056
	Day2 Δ sAA	-0.088
	Day1 Δ sIgA	-0.041
	Day2 Δ sIgA	-0.070

Supplementary Table 4. Adjusted R-square values for multiple regression model with predictors: BMI, Ethnicity, Age, and Gender. Δ sAA / Δ sIgA was derived by subtracting sAA am/ sIgA am from sAA pm/ sIgA pm values.

Predictors	Dependent Variables	Adjusted R square
BMI, Ethnicity, Age (months), Gender	Day1 sAA am	0.038
	Day1 sAA pm	0.006
	Day2 sAA am	0.071
	Day2 sAA pm	0.020
	Day1 sIgA am	0.003
	Day1 sIgA pm	-0.030
	Day2 sIgA am	0.091
	Day2 sIgA pm	-0.014
	Day1 Δ sAA	-0.031
	Day2 Δ sAA	0.042
	Day1 Δ sIgA	-0.002
	Day2 Δ sIgA	0.072

Supplementary Table 5. sIgA and sAA expression (in pg/ml) in morning and evening on two days in children who were reported by the parents to have eczema (N=16)

	**Day1**		**Day2**		**Day1**		**Day2**	
	**sIgA AM**	**sIgA PM**	**sIgA AM**	**sIgA PM**	**sAA AM**	**sAA PM**	**sAA AM**	**sAA PM**
1	441750.3	31878.6	312800.6	32238.1	31.1	121.7	53.6	88.6
2	173858.4	33917.8	106326.3	45107.6	45.7	78.3	54.2	119.9
3	60966.6	27719.8	240224.3	57723.7	37.5	98.5	31.1	139.7

4	471842.6	18661.2	23159.8	219402.2	48.7	89.1	184.8	6.4
5	634003.9	32775.6	256196.4	66746.8	32.9	52.3	40.3	95.5
6	269030.0	19599.9	89767.2	27931.6	111.9	38.8	49.1	200.5
7	30361.9	11647.3	40960.9	14607.8	113.9	22.0	64.4	167.1
8	22382.2	18178.5	44312.9	19890.5	45.5	110.3	60.9	85.7
9	73242.2	38934.6	117511.2	35171.2	32.3	43.9	40.5	108.9
10	82396.7	98685.3	69161.9	27739.3	31.8	54.4	41.3	34.1
11	60167.3	29475.2	45743.3	266443.1	89.3	164.0	124.2	31.8
12	36593.3	37750.3	197987.2	35846.3	67.7	15.9	75.0	44.2
13	109652.9	16673.6	157213.7		22.6	124.3	75.2	
14	288347.0	231605.9	46254.1	78005.9	26.9	56.2	17.5	
15	244683.0	68942.4	625482.2	583324.7	19.0	46.9	54.4	56.0
16	53889.5	26185.7	51037.2	54029.2	68.3	87.8	41.3	105.5
**Mean**	190823.0	46414.5	151508.7	104280.5	51.6	75.3	63.0	91.7
**Stdev**	186176.1	53941.7	154493.5	151380.9	30.3	41.1	40.3	54.7
*	*							

* - significant difference: Mann Whitney test was performed and significance was defined if p<0.05

Supplementary Table 6. sIgA and sAA expression (in pg/ml) in morning and evening on two days in children who were reported by the parents to have asthma (N=17)

	**Day1**		**Day2**		**Day1**		**Day2**	
	**sIgA AM**	**sIgA PM**	**sIgA AM**	**sIgA PM**	**sAA AM**	**sAA PM**	**sAA AM**	**sAA PM**
1	173858.4	33917.8	106326.3	45107.6	45.7	78.3	54.2	119.9
2	60966.6	27719.8	240224.3	57723.7	37.5	98.5	31.1	139.7
3	79095.3	39439.9	49555.2	39478.7	97.5	36.2	22.6	42.1
4	34001.9	53547.9	74025.2	28043.1	38.3	68.3	46.4	62.4
5	244571.7	104534.7	123787.1	62144.0	17.0	244.6	44.4	90.9
6	269030.0	19599.9	89767.2	27931.6	111.9	38.8	49.1	200.5
7	19086.8	15748.3	220212.7	30419.0	59.0	253.4	78.0	184.0
8	44410.5	38516.4	52975.0	102113.5	10.8	37.4		29.0
9	73242.2	38934.6	117511.2	35171.2	32.3	43.9	40.5	108.9
10	125232.3	61857.0	192941.7	69163.2	67.7	100.1	101.7	166.9
11	70722.1	11502.0	71674.8	65014.2	51.4	59.1	38.3	39.2
12	36593.3	37750.3	197987.2	35846.3	67.7	15.9	75.0	44.2
13	199112.5	59924.3		98990.6	27.5	64.2	34.2	80.4
14	111900.9	54523.2	510316.7	154907.0	72.6	11.0		68.3
15	30755.5	35485.9	221466.2	39402.1	107.8	82.6	67.8	66.7
16	391389.6	125243.0	329255.6	111648.2	85.8	167.9	75.0	146.6
17	37658.3	34409.1	75032.8	17198.3	104.4	120.7	122.5	155.1
**Mean**	117742.8	46626.7	167066.2	60017.8	60.9	89.5	58.7	102.7
**Stdev**	104676.1	29599.8	122183.8	37174.6	32.2	71.7	27.8	54.8

*	*	*		*

* - significant difference: Mann Whitney test was performed and significance was defined if p<0.05

Supplementary Table 7. sIgA and sAA expression (in pg/ml) in morning and evening on two days in children who were reported by the parents to have food allergy (N=7)

	Day1		Day2		Day1		Day2	
	sIgA AM	sIgA PM	sIgA AM	sIgA PM	sAA AM	sAA PM	sAA AM	sAA PM
1	441750.3	31878.6	312800.6	32238.1	31.1	121.7	53.6	88.6
2	173858.4	33917.8	106326.3	45107.6	45.7	78.3	54.2	119.9
3	103528.4	81284.6	144750.4	71889.8	119.3	44.6	37.0	24.6
4	34255.3	71818.2	75064.6	56442.0	101.9	28.8	79.0	58.0
5	32459.6	18744.3	43967.4	30085.8	36.9	32.6	22.4	49.6
6	44410.5	38516.4	52975.0	102113.5	10.8	37.4		29.0
7	441750.3	31878.6	312800.6	32238.1	31.1	121.7	53.6	88.6
Mean	138377.1	46026.6	122647.4	56312.8	57.6	57.2	49.2	61.6
Stdev	158365.5	24721.9	100221.8	27325.3	43	36.3	21.2	36.7
*								

* - significant difference: Mann Whitney test was performed and significance was defined if p<0.05

Supplementary Table 8. Mean morning and evening values of sIgA and sAA expression for healthy children and children with parent-reported allergy.

mean value	Children with parent-reported allergy	Healthy children	P value, Mann-Whitney
sIgA AM day 1, ug/ml	155837.2	150898.9	0.9836
sIgA PM day1, ug/ml	46447.3 **	55570.4 ##	0.1969
sIgA AM day 2, ug/ml	153502.2	153133.5	0.2022
sIgA PM day 2, ug/ml	76904.9 **	70583.0 ##	0.6300
sAA AM day 1, U/ml	56.6	68.0	0.4654
sAA PM day 1, U/ml	78.7	92.0 #	0.3960
sAA AM day2, U/ml	59.3	69.6	0.7837
sAA PM day2, U/ml	91.9 *	82.9 #	0.3532

* - indicate significant difference between AM and PM values for Children with parent-reported allergy, based on Mann-Whitney p<0.05 (*) and p<0.001 (**)

# - indicate significant difference between AM and PM values for Healthy children, based on Mann-Whitney p<0.05 (#) and p<0.001 (##)
